# A Case Series: Adjunctive Treatment of Major Depressive Disorder in the Geriatric Population with the Methylphenidate Patch

**DOI:** 10.1155/2019/2890913

**Published:** 2019-12-08

**Authors:** Kamal Patel, Ashley Collins, Suzanne Holroyd, Andre Benja Lamyaithong

**Affiliations:** Department of Psychiatry & Behavioral Medicine, Joan C. Edwards School of Medicine, Marshall University, Huntington, WV, USA

## Abstract

Major depressive disorder can affect anyone regardless of age. In geriatric populations depression is often overlooked and untreated, which subsequently may lead to serious consequences. Almost one third of elderly patients with depression fail to respond to initial treatment and require adjunctive treatment. Methylphenidate is one such option, which is seldom used in the geriatric population to treat depression despite reports of improvement in symptoms of mood within a brief period of time. Methylphenidate is also available in a patch formulation that can be used in patient's nonadherent to the medication, which is reported to be an issue in as many as 75% of the geriatric population. Here we present three geriatric patients who were diagnosed with recurrent severe major depressive disorder without psychotic features. The three patients responded well with methylphenidate as adjunctive treatment to conventional antidepressants.

## 1. Introduction

Depression, which is one of the most common mental health problem, is found to affect more than two million of the 34 million Americans age 65 and older [1]. Further, the geriatric population ages 65 and older, which comprises only 13% of the US population, accounts for 20% of all suicide deaths [1].

Symptoms of depression can affect the geriatric population's ability to participate in rehabilitation and other aspects of care leading to an increase in adverse outcomes [2]. The geriatric population can present treatment challenges. Approximately 25–30% of patients fail to respond to initial treatment [3], which leads requiring additional treatment. One strategy includes use of methylphenidate. Methylphenidate is one medication that has been recommended for use to augment antidepressants in geriatric depression [4].

Nonadherence can pose another challenge in treating geriatric population. It has been estimated that 40–75% percent of elderly patients are noncompliant with medication [5].

One possible method of reducing patient nonadherence may be alternative administration routes, such as the transdermal patch formulation, which is available for methylphenidate in four doses, which include 10 mg, 15 mg, 20 mg, and 30 mg. The methylphenidate patch is delivered over nine hours. The acrylic adhesive contains the methylphenidate, which is applied on the skin and thus delivers medicine into the skin and subsequently into bloodstream. Onset of action is approximately two hours and has half-life elimination of three to four hours [6]. Peak plasma concentrations of methylphenidate is reached approximately about eight hours after patch application. The absorption through patch is steadier, thus providing consistent exposure during dosing and prevent adverse events. Multiple dosing with the patch despite over 9 hour wear time has shown to increase serum level methylphenidate [7].

## 2. Case Presentations

### 2.1. Case 1

The patient was a 94-year-old male with a diagnosis of major depressive disorder, recurrent, severe, without psychotic features as well as major neurocognitive disorder, severe, likely vascular type, with behavioral disturbance. He presented with depressed mood, tearfulness, anxiety, decreased oral intake, hopelessness, and passive suicidal ideations. The patient had been refusing medications frequently. He was started on oral venlafaxine and mirtazapine; however, he was not consistently getting these medications as he was often refusing. The venlafaxine was increased to 100 mg twice daily and switched to a pluronic lecithin organogel (PLO) cream. Although there was some improvement in mood with the venlafaxine, the patient was still experiencing symptoms of intermittent tearfulness, low energy, and decreased oral intake. Given the oral medication refusals, the patient was started on the methylphenidate 10 mg patch for 9 hours daily. After two weeks, the patient started showing remarkable improvement. He was more alert, more responsive, and oral intake increased. His affect was brighter and he was smiling frequently. He no longer voiced any passive suicidal ideations.

### 2.2. Case 2

The patient was an 81-year-old female with a diagnosis of major depressive disorder, recurrent, severe, without psychotic features as well as major neurocognitive disorder, severe, likely vascular type, with behavioral disturbance. She presented with depressed mood, tearfulness, low energy, decreased oral intake, and refusal of medications as well. Oral venlafaxine and mirtazapine were discontinued as she had been refusing oral medications. She was started on venlafaxine PLO cream and titrated up to 100 mg twice daily. The patient had partial improvement in her mood symptoms with venlafaxine. At that time, the methylphenidate patch 10 mg for 9 hours daily was started. Approximately one week later, the patient was more interactive with staff and co-residents on the unit. She was more alert and awake throughout the day. She was more pleasant, smiling during interactions, and she was no longer irritable.

### 2.3. Case 3

The patient was a 69-year-old male with a diagnosis of major depressive disorder, recurrent, severe, without psychotic features. He presented with a depressed mood and apathy. He endorsed hopelessness stating his future looked “dim”. The patient had been resistant to self-care and care from staff members. Appetite had been decreased and he lost approximately 16 kgs over one year. Energy level was decreased. He also had significant speech latency and psychomotor retardation. He was initially switched from sertraline to venlafaxine, which was titrated up to 300 mg daily. Bupropion extended release was started and titrated to 300 mg daily to augment the venlafaxine. The patient showed minimal improvement but was still depressed and withdrawn. At that point oral methylphenidate was added. The patient subsequently started becoming nonadherent with medications. Venlafaxine was switched the PLO formulation. Methylphenidate was switched to the patch formulation, which was titrated up to a dose of 30 mg for 9 hours daily. The patient responded to this dose without any adverse effects. The patient has since become compliant with medications and has now been transitioned back to oral venlafaxine and oral methylphenidate, which was increased to 40 mg daily. The patient was significantly improved with regard to mood, affect and compliance with care.

## 3. Discussion

This case series presents three geriatric patients with a history of Major depressive disorder, recurrent, severe, without psychotic feature, who were successfully treated with an antidepressant along with addition of methylphenidate. Two of the cases were diagnosed with major neurocognitive disorder, severe, likely vascular type, with behavioral disturbance in addition to major depressive disorder. Given that all the patients had histories of nonadherence, PLO formulation of venlafaxine and methylphenidate patch were used. All three patients showed significant improvement with less depressive symptoms within a short period of time. Two of the patients showed significant improvement on the 10 mg methylphenidate patch and tolerated this dosage well without any adverse events. The third patient needed to be titrated up to the 30 mg methylphenidate patch. The third patient eventually started taking oral medications and was switched to the oral methylphenidate extended release formulation. The dose was increased to 40 mg before noticing positive response to his depression. The patient tolerated the dosage well without any adverse events. The patients reported as case 1 and 3 were on the methylphenidate patch for five months, whereas the patient in case 2 was on the medication for four months. No significant changes in vital signs were noted in any of the three patients neither before nor after administration of methylphenidate patch or oral formulation.

Previous studies have shown that methylphenidate used in the geriatric population is effective in the treatment of mood and cognition. In one study withdrawn, apathetic geriatric patients were randomized into treatment with 20 mg of methylphenidate (*n* = 25) or placebo (*n* = 19). Significant improvement was noted in the group receiving methylphenidate [8]. The most notable improvement was in attention, interest, self-esteem, and involvement, which was also noted in our patients [8]. Another study reported improvement in depression, mini mental status examination score, and functional status among elderly patients (*n* = 23) treated with methylphenidate [9].

Per the literature, many geriatric patients have been shown to tolerate 10–30 mg of oral methylphenidate [8, 10]. Methylphenidate has been shown to have a very low risk of adverse events and appears to be at least as safe and well tolerated as standard antidepressants [2]. The most common notable adverse events reported with use of methylphenidate include agitation, restlessness, sinus tachycardia, palpitations, delirium, hypertension, and insomnia [2]. None of the patients presented in this case series had any notable adverse event including the third case who received oral methylphenidate extended release 40 mg daily.

Recognizing and treating depression in the geriatric population is of utmost importance given its poor outcome when not diagnosed or treated. Cole et al. [11] reviewed studies involving geriatric patients with depression treated in elderly community and primary care. In a meta-analysis of the patients who received treatment and was followed-up after two years only 33% were doing well, whereas 33% were still depressed, and 21% had passed away [11]. Untreated depression has been shown to worsen clinical manifestations of comorbid chronic medical conditions such as hypertension and diabetes mellitus leading to poorer outcomes [12].

In conclusion, methylphenidate is an effective and well tolerated adjunctive medication for depression along with antidepressants in elderly populations. The present case reports with patch formulation of methylphenidate show the utility this may add to treatment for apathetic geriatric patients as noted by other studies as well [8, 9]. The patch is a promising treatment modality for patients who are nonadherent with oral medications. The above cases demonstrate significant response to the methylphenidate patch. Without this alternative formulation, the choices of medications for augmenting depression treatment are limited. Although there is abundant literature on oral methylphenidate, there is need for further research on the use of the methylphenidate patch formulation as adjunctive treatment of major depressive disorder.
